# Oral–Gut Microbial Crosstalk and Therapeutic Applications of Bacterial Extracellular Vesicles

**DOI:** 10.3390/biom16010026

**Published:** 2025-12-24

**Authors:** Wenmei Fu, Ninghan Yang, Jiale Yan, Bing Han, Qin Niu, Zhengyu Li, Rushui Bai, Tingting Yu

**Affiliations:** 1 Department of Orthodontics, Peking University School and Hospital of Stomatology & National Center for Stomatology & National Clinical Research Center for Oral Diseases, Beijing 100081, China; 2310303131@stu.pku.edu.cn (W.F.); 2310303143@stu.pku.edu.cn (N.Y.); 1810303127@pku.edu.cn (J.Y.); kqbinghan@bjmu.edu.cn (B.H.); 2511110639@stu.pku.edu.cn (Q.N.); 2511110626@bjmu.edu.cn (Z.L.); 2 First Clinical Division, Peking University School and Hospital of Stomatology & National Center for Stomatology & National Clinical Research Center for Oral Diseases, Beijing 100081, China

**Keywords:** oral–gut crosstalk, microbiome, bacterial extracellular vesicles, periodontitis, inflammatory bowel disease

## Abstract

With the accelerating trend of global population aging, oral and gut diseases are imposing a rising socioeconomic burden, both of which have well-known connections to microbial dysbiosis. As the gateway to the human body, the oral cavity exhibits close interactions with the gastrointestinal tract, which includes translocation of bacteria and bacterial extracellular vesicles (BEVs), as well as intermucosal immunity and neural signaling. These oral–gut crosstalk pathways play vital roles in the pathogenesis and progression of oral diseases, such as periodontitis, and gut diseases, such as inflammatory bowel disease (IBD). Focusing on periodontitis and IBD as representative conditions, this review summarizes current understanding of the oral–gut crosstalk and underlying mechanisms. Among diverse interactions, we emphasize BEVs as effective trans-barrier mediators and their therapeutic potentials during oral–gut crosstalk. Beneficial BEVs, notably those from *Akkermansia muciniphila* (*Akk*), exert various probiotic roles, including modulating microbial homeostasis, promoting tissue repair and alleviating inflammation, thereby shedding light on the prevention and treatment of oral and gut diseases, even systemic disorders.

## 1. Introduction

Population aging contributes to a growing socioeconomic burden posed by oral and gut diseases. Oral diseases, represented by dental caries and periodontitis, affect nearly 3.5 billion individuals worldwide. The oral hygiene status of Chinese population represents a significant public health challenge, with the prevalence of periodontitis among middle-aged and older adults exceeding 90%, which is far higher than that of chronic non-communicable diseases such as cardiovascular disorders and cancer [1]. Concurrently, inflammatory bowel disease (IBD) and other chronic gut disorders have emerged as pressing public health concerns worldwide, severely compromising patients’ quality of life. Notably, the most rapid increase in prevalence over the past decade has been observed in newly industrialized countries, posing significant challenges to aging populations [2,3,4]. Traditional therapies for oral and gut diseases mainly focus on local symptom alleviation while overlooking systemic inter-organ or host-microbe interactions, which contribute to high recurrence rates and drug resistance. The human body is colonized by over 100 trillion symbiotic microorganisms, collectively referred to as the human microbiome. Recent studies have highlighted the profound potential of microbial dysbiosis in the pathogenesis and progression of oral and gut disorders [5,6,7,8,9,10]. Interventions targeting microbiota have achieved promising therapeutic results and the markets for microbiota-based therapies are expanding rapidly. Therefore, gaining deeper insights into host-microbiota interaction is crucial for unlocking further therapeutic potential of these strategies [11,12].

**Figure 1:**
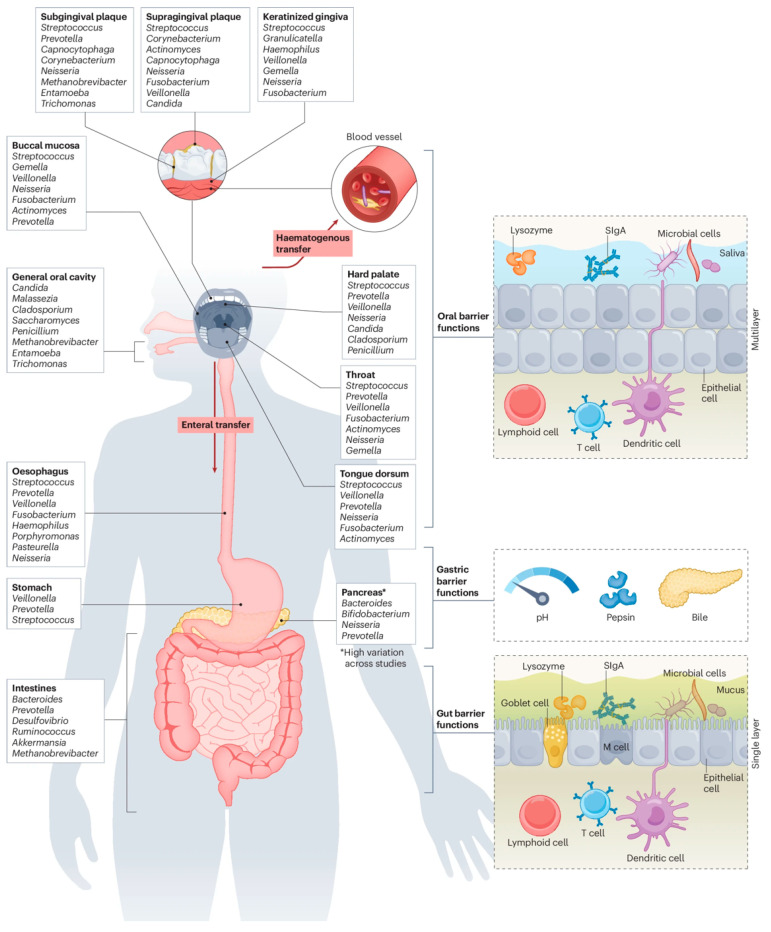
Oral–gut microbial continuity and defense barriers. The gastrointestinal tract is a continuous mucosal tube from the mouth to the anus, featuring site-specific but interconnected microbial communities. Despite robust physical and immune barriers, oral bacteria can migrate to the gut via hematogenous or enteral routes, especially when barrier function is impaired. The left panel shows the main microbial niches and translocation routes, while the right panel summarizes the key physical and immune defense mechanisms in the oral cavity, stomach, and gut. Reproduced from Kunath et al. [14], Springer Nature, with permission.

The oral cavity and gut represent the largest microbial habitats, playing important roles in microbiota-related diseases [13]. It is well established that various oral disorders, including gingivitis, periodontitis, dental caries and oral cancer, have been associated with oral dysbiosis [14]. As continuous components of the digestive tract, the oral cavity and gut are intimately linked through anatomical, microbial and immunological connections [15]. In recent years, the concepts of the “oral–gut axis” and “gut–oral axis” have received growing attention, emphasizing the dynamic inter-organ interplay (Figure 1). Oral bacteria can translocate to the gastrointestinal tract via hematogenous and enteral routes, potentially exacerbating various gut diseases. Patients with gut disorders exhibit characteristic enrichment of oral pathogens in intestinal contents and mucosal tissues, and conversely, the oral cavity is a common site for extraintestinal manifestations of gut diseases such as IBD, with a notably increased prevalence of periodontitis among IBD patients compared with non-IBD controls [16,17]. Understanding the bidirectional oral–gut microbial crosstalk is vital for elucidating the systemic impact of microbiota on human health and disease.

Dynamic microbial involvement in oral and gut diseases indicates the promising potential of microbiota-based strategies for disease prevention and treatment. Probiotics exert beneficial effects on alleviating oral and intestinal inflammation by regulating microbial homeostasis and enhancing the mucosal barrier, thereby contributing to the maintenance of human health [18,19]. Yet, probiotics have inherent limitations—bacterial viability is usually compromised by physiological barriers, and translocated bacteria can be impeded by gut mucosal defenses and be outcompeted by resident gut microbiota [20]. To overcome limitations of live bacteria, bacterial extracellular vesicles (BEVs) derived from probiotics have gained increasing interest, due to their nanoscale size, superior biocompatibility, and reduced pathogenic risk [21]. BEVs from *Akkermansia muciniphila* (*Akk*), exhibit capacities to mitigate inflammation, and even enhance immunotherapy by targeting the tumor microenvironment [22]. In contrast, pathogenic BEVs are more permeable across physiological barriers and carry pathogenic components such as lipopolysaccharides (LPS) and bacterial DNA, thus playing crucial roles in bacterial colonization and virulence factor dissemination [23]. Thus, BEVs have emerged as a promising alternative or complementary strategy for microbiota-based interventions.

This review aims to summarize the current understanding of oral–gut microbial crosstalk, especially gut microbial changes induced by oral disorders and oral dysbiosis that occur along with gut diseases. We emphasize the roles of BEVs in oral–gut crosstalk, particularly, to shed light on the diagnosis, prevention and treatment of chronic oral and gut diseases.

## 2. Oral–Gut Microbial Interactions in Diseases

The oral cavity and gut have continuity in anatomy, harbor the most abundant microbiota in the human body and influence each other through dynamic routes. High-throughput sequencing analysis of microbial communities across different digestive tract regions has revealed four distinct community types across ten digestive tract sites. These include Group 1 (oral mucosa, keratinized gingiva, hard palate), Group 2 (saliva, tongue, tonsils, throat), Group 3 (subgingival and supragingival plaque), and Group 4 (feces). Each group exhibits distinct microbial compositions: Bacillota dominate Group 1, Bacteroidota and Fusobacteriota are prominent in Group 2, Actinomycetota increase in Group 3, and Bacteroidota prevail in Group 4. Despite their close proximity, these sites demonstrate a degree of taxonomic continuity in microbial distribution, yet their community structures differ significantly in relative abundance and composition [24].

### 2.1. Gut Dysbiosis Under Periodontitis

Periodontitis is driven by the synergistic interactions and dysbiosis of polymicrobial communities, typically associated with a shift in the microbial community from a healthy state to a dysbiotic state [25,26,27]. In periodontitis patients, the microbial diversity of supragingival plaque is increased [28,29,30]. Periodontitis not only affects the oral microenvironment but is increasingly recognized as being closely associated with systemic health. Periodontal pathogens such as *Porphyromonas gingivalis* (*Pg*) and *Fusobacterium nucleatum* (*Fn*) can promote inflammatory responses, impair the integrity of the intestinal barrier, and impact various systemic diseases via the oral–gut-systemic axis. For example, periodontitis is closely associated with non-alcoholic fatty liver disease [28,31,32], multiple types of cancer (e.g., oral squamous cell carcinoma and colorectal cancer (CRC)) [33,34,35,36,37], cardiovascular diseases, Alzheimer’s disease [38,39], and diabetes mellitus [40]. Periodontal pathogens contribute to pathological processes, including hepatic lipid accumulation, atherosclerotic plaque formation, and insulin resistance. Although the precise causal relationships and underlying molecular mechanisms remain to be fully elucidated, substantial evidence have indicated that the oral–gut crosstalk is one of the major pathways through which periodontitis impacts systemic health.

**Figure 2:**
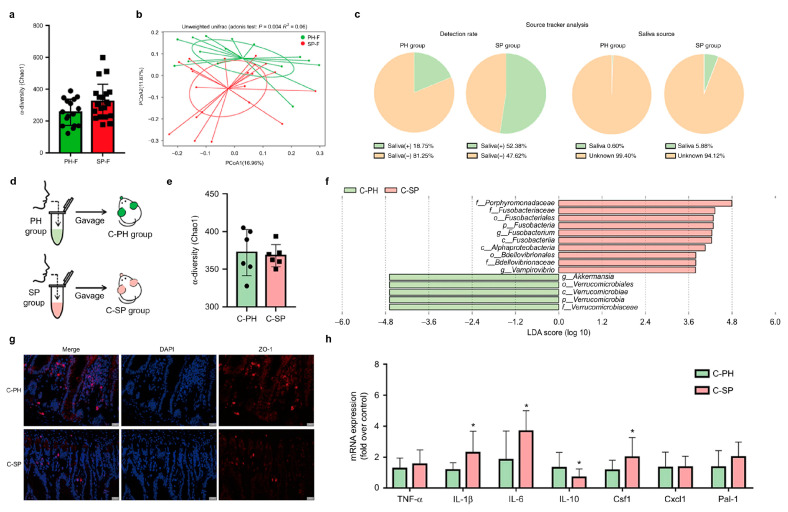
Oral-derived microbiota translocate to the gut under periodontitis, reshaping the gut microbiome, and altering intestinal barrier and inflammation. (**a**–**c**) Human data. (**a**) α diversity-Chao1 of the salivary microbiota in periodontitis patients (PH) versus healthy controls (SP). Data were expressed as the mean  ±  SD. Statistical significance was determined through the Wilcoxon test. (**b**) Principal coordinates analysis (PCoA) shows separate clustering. (**c**) The Source Tracker analysis shows saliva-derived microbiota in the fecal samples. (**d**–**f**) Mouse experiment. (**d**) Schematic: mice were gavaged with saliva from PH or SP donors to generate C-PH and C-SP groups. (**e**) α diversity-Chao1 of the fecal microbiota. Data were expressed as the mean  ±  SD. Statistical significance was determined using the Wilcoxon test. (**f**) The LDA graph shows the taxonomic differences in the C-PH and C-SP groups. (**g**,**h**) Effects on the intestinal barrier and inflammation. (**g**) Representative images of immunofluorescence-stained sections of proximal colon tissue targeting ZO-1 (red) and DAPI (blue). Scale bar, 20 µm. (**h**) Relative mRNA expression of intestinal inflammatory markers. Statistical significance was determined using the *t*-test. (* *p* < 0.05) The phylum names in this figure are presented as originally published. In this review, the modern nomenclature is used, where Fusobacteria is now referred to as Fusobacteriota [48]. Adapted from Bao et al. [44], licensed under CC BY 4.0.

Several studies collected fecal samples from patients with periodontitis and healthy individuals. These studies found that the β diversity of the gut microbiota in periodontitis patients is significantly altered: the relative abundance of pathogenic bacteria increases, while that of beneficial bacteria decreases. Meanwhile, abnormal colonization of oral-origin pathogens or commensal bacteria occurs in the gut of periodontitis patients, as shown in Figure 2. Compared with healthy controls, higher abundances of the following were detected in the fecal samples of periodontitis patients: the phylum Bacteroidota, the family Lachnospiraceae and the genera *Faecalibacterium*, *Clostridium*, *Fusobacterium*, *Streptococcus* and *Porphyromonas* [15,41,42,43,44,45]. It is known that probiotics can produce short-chain fatty acids (SCFAs), which help maintain the integrity of the intestinal barrier and inhibit inflammatory responses. They also play a role in regulating the gut immune system and contributing to the balance of the gut microbiota. Their reduction may further exacerbate the inflammatory status of the gut. Animal experiments validated that after inducing chronic periodontitis through ligature placement [46] or inducing chronic apical periodontitis by exposing the pulp chamber and inserting a cotton pellet containing *Pg* [47], subsequent 16S rRNA gene sequencing of gut microbiota showed a significant reduction in *Turicibacter*, and enrichment of Bacteroidota. After gavaging saliva from human periodontitis patients into healthy mice, gut microbiome analysis confirmed that salivary bacteria could persist in the gut for at least 24 h, supporting a mechanistic basis for ectopic colonization of oral microbiota in the gut. Histological and gene expression analyses were also performed to assess pathological changes in intestinal tissues, validating that the enrichment of oral-origin bacteria in the gut may promote inflammation or disrupt intestinal barrier function, thereby exacerbating disease. This is evidenced by significantly increased mRNA expression levels of inflammatory cytokines (e.g., IL-1β, IL-6) and significantly decreased expression of the anti-inflammatory cytokine IL-10, as well as reduced expression of the tight junction protein zonula occludens-1 (ZO-1) and decreased numbers of mucus-producing cells [44].

Moreover, studies have shown that periodontal treatment is associated with significant changes in the gut microbiota. Treating periodontitis can partially restore the homeostasis of gut microbiota. In the feces of periodontitis patients before treatment, the Bacillota/Bacteroidota ratio was significantly lower than that of healthy controls. At the genus level, the detection frequency of *Bacteroides, Butyricicoccus*, *Clostridium*, *Escherichia*, *Blautia*, *Faecalibacterium*, *Lactobacillus*, and Lachnospiraceae, among others, was higher than that in healthy controls [14]. In contrast, *Lactobacillus* was the only genus more abundant in healthy controls. After treatment, a significant reduction was observed in the abundance of multiple genera, notably *Bacteroides*, *Butyricicoccus*, *Blautia*, *Faecalibacterium*, *Lactobacillus*, and members of the Lachnospiraceae family, while the Bacillota/Bacteroidota ratio significantly increased. Overall, the gut microbiota profile of patients with periodontitis after treatment became similar to that of healthy controls, providing indirect evidence that periodontitis has a significant impact on the gut microbiota [41].

### 2.2. Oral Impact of IBD

IBD is primarily categorized into two major types: Crohn’s disease (CD), which can affect any part of the gastrointestinal tract with transmural inflammation [49], and ulcerative colitis (UC), which is characterized by continuous mucosal inflammation confined to the colon [50,51]. Studies have shown that the gut microbiota diversity in IBD patients is significantly lower than in healthy individuals, with beneficial bacteria reduced and pathogenic bacteria increased. In human studies, the relative abundance of oral bacteria in the feces of IBD patients was significantly higher than that of healthy controls, while the total microbial load in the gut was significantly lower. The similarity between oral and gut microbiota was also markedly higher in IBD patients compared to healthy controls.

Intestinal inflammation is closely associated with alterations in the oral environment. IBD patients exhibit marked changes in the oral environment, characterized by increased oral inflammation, disruption of the oral microbial community structure and metabolic dysfunction. Clinically, studies have found that patients with CD are more prone to extra-intestinal oral lesions, such as cobblestoning and aphthous ulcers. Moreover, the prevalence of periodontitis is significantly increased in individuals with IBD, as evidenced by more severe probing depth, bleeding index, and attachment loss [52,53]. Similarly, pyostomatitis vegetans is strongly correlated with IBD activity [54]. IBD patients frequently exhibit compromised oral mucosal barriers due to immune dysregulation. Overactivation of the Th17 pathway and the release of pro-inflammatory cytokines such as TNF-α and IL-6 trigger recurrent aphthous ulcers and mucosal erosions [55,56]. The specific mechanisms will be described in Section 3. Additionally, alterations in salivary composition exacerbate oral microenvironmental imbalance. Elevated levels of calprotectin and reduced antioxidant enzyme activity in the saliva of IBD patients intensify oxidative stress, while acidic salivary pH creates a favorable niche for pathogenic bacterial proliferation [57].

As the oral environment changes, IBD patients exhibit marked dysbiosis of the oral microbiota. Distinct oral microbial profiles are observed between individuals with and without IBD. Specifically, IBD patients demonstrate a marked enrichment of Bacteroidota, and a reduction in the abundance of Fusobacteriota and Lachnospiraceae [52,53,58,59]. The specific microbial alterations are summarized in Table 1. It is noteworthy that in different studies, the trends in the abundance changes in Streptococcaceae and Prevotella are not consistent. Studies indicate that the extent of oral microbial dysbiosis is positively associated with IBD severity, and can be partially restored following treatment [60]. Oral microbial dysbiosis serves not only as an oral hallmark of enteritis but also as a key driver of systemic inflammatory progression [61]. IBD patients show decreased oral microbial diversity and abnormal overgrowth of opportunistic pathogens. The accumulation of *Fn* in periodontal pockets not only aggravates local inflammation via the Toll-like receptor 4 (TLR4)/NF-κB pathway but can also translocate to the gut, directly impairing the intestinal epithelial barrier [17]. Concurrently, *Prevotella* overgrowth correlates positively with ulcer severity, as its lipopolysaccharides induce epithelial apoptosis. The depletion of commensal genera such as *Streptococcus* and *Rothia* weakens colonization resistance, facilitating pathogen dominance. Poor oral hygiene amplifies this dysbiosis; dental plaque accumulation and periodontal pocket formation increase pathogenic bacterial load, which exacerbates intestinal inflammation through a “swallow-colonization” mechanism [62,63]. Critically, microbial metabolites (e.g., butyrate) from the dysregulated oral microbiota can systemically influence intestinal barrier function via circulation [59].

Nutritional metabolic disturbances play a critical role in this process. Deficiencies in vitamin B12 and iron, common in IBD patients, can directly impair lingual papillae and gustatory nerve function. These deficiencies further disrupt the vitamin-synthesizing capacity of the oral microbiota, perpetuating a vicious cycle manifested as glossitis, dysgeusia, and delayed mucosal repair [64]. Collectively, these physicochemical alterations establish the pathological basis of oral lesions and may, in turn, exacerbate intestinal inflammation via the oral–gut crosstalk [65].

**Table 1:** Microbial changes in Periodontitis vs. IBD.

Disease	Classification	Abundance	Methods	Objects of Study	References
Periodontitis	Lachnospiraceae	Increased	16S rRNA sequencing	Fecal samples from patients with periodontitis	Baima, G. et al. [41] Bao, Jun. et al. [44]
	Bacteroidota	Increased	16S rRNA sequencing	Fecal samples from patients with periodontitis	Baima, G. et al. [41] Arimatsu, K. et al. [66]
			16S rRNA sequencing	Ileal contents from *Pg*–treated mice	Sasaki, N. et al. [28]
	*Faecalibacterium*	Increased	16S rRNA sequencing	Fecal samples from patients with periodontitis	Baima, G. et al. [41]
	*Clostridium*	Increased	16S rRNA sequencing	Fecal samples from patients with periodontitis	Bao, Jun. et al. [44] Kamer, A. R. et al. [67]
	*Fusobacterium*	Increased	High-throughput whole metagenome sequencing	Fecal samples from patients with periodontitis	Baima, G. et al. [68]
	*Streptococcus*	Decreased	16S rRNA sequencing	Fecal samples from patients with periodontitis	Bao, Jun. et al. [44]
			High-throughput whole metagenome sequencing	Fecal samples from patients with periodontitis	Baima, G. et al. [68]
	*Porphyromonas*	Increased	16S rRNA sequencing	Fecal samples from patients with periodontitis	Bao, Jun. et al. [44]
	*Lactobacillus*	Decreased	16S rRNA sequencing	Fecal samples from patients with periodontitis	Baima, G. et al. [41] Kamer, A. R. et al. [67]
			High-throughput whole metagenome sequencing	Fecal samples from patients with periodontitis	Baima, G. et al. [68]
	*Faecalibacterium*	Decreased	High-throughput whole metagenome sequencing	Fecal samples from patients with periodontitis	Baima, G. et al. [68]
	*Bacillota*	Decreased	16S rRNA sequencing	Ileal contents from *Pg*–treated mice	Sasaki, N. et al. [28]
	*Turicibacter*	Decreased	16S rRNA sequencing	Oral *Aggregatibacter actinomycetemcomitans* mice	Komazaki, R. et al. [69]
IBD	Bacteroidota	Increased	16S rRNA sequencing	Saliva from patients with IBD	Said, H. S. et al. [58] Docktor, M. J. et al. [70] Hu, S. et al. [53]
	*Prevotella*	Increased	16S rRNA sequencing	Saliva from patients with IBD	Said, H. S. et al. [58]
		Decreased	16S rRNA sequencing	Saliva from patients with IBD	Xun, Z. et al. [71]
	Veillonellaceae	Increased	16S rRNA sequencing	Saliva from patients with IBD	Xun, Z. et al. [71]
	Fusobacteriota	Decreased	16S rRNA sequencing	Tongue and buccal mucosal brushings from patients with IBD	Docktor, M. J. et al. [70]
	Lachnospiraceae	Decreased	16S rRNA sequencing	Saliva from patients with IBD	Xun, Z. et al. [71]
	Streptococcaceae	Increased	16S rRNA sequencing	Saliva from patients with IBD	Xun, Z. et al. [71]
		Decreased	16S rRNA sequencing	Saliva from patients with IBD	Said, H. S. et al. [58]
	Enterobacteriaceae	Increased	16S rRNA sequencing	Saliva from patients with IBD	Xun, Z. et al. [71]

IBD: Inflammatory bowel disease; *Pg*: *Porphyromonas gingivalis.*

## 3. Underlying Mechanisms of Oral–Gut Microbial Crosstalk

The oral cavity and the intestinal tract, as the two largest microbial colonization areas in the human body, share similar environmental and structural characteristics, including the colonized bacterial communities, the epithelial tissue barriers, and the immune defenses. Oral–gut microbial crosstalk involves complex direct and indirect mechanisms. Direct mechanisms refer to oral microbiota migration and gut colonization, while indirect mechanisms include circulatory processes mediated by immune cells and cytokines (exemplified by the oral–gut–liver axis) and neuroimmune signaling cascades (exemplified by the oral–gut–brain axis).

**Figure 3:**
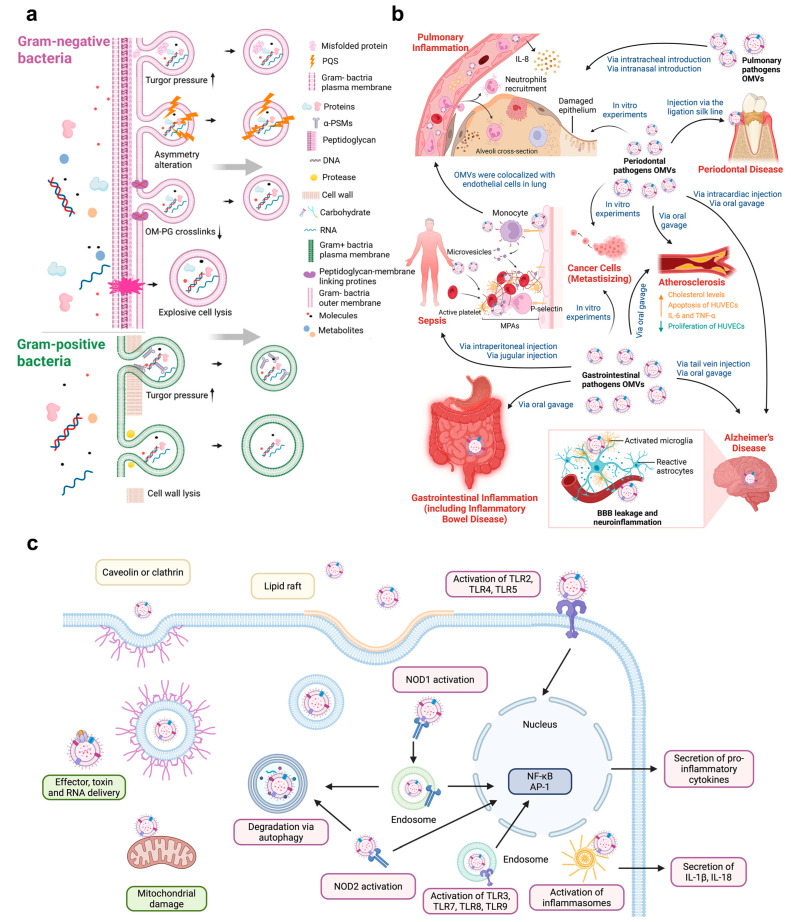
The lifecycle and pathogenic mechanisms of bacterial extracellular vesicles (BEVs). (**a**) Biogenesis and cargo packaging. There are three major types of vesicles produced by Gram-negative bacteria: the classic outer membrane vesicles (OMVs) produced by the bubbling of living cells, outer–inner membrane vesicles (OIMVs) and explosive outer membrane vesicles (EOMVs) produced by cell rupture triggered by endolysins, and OIMVs formed by the transient degradation of peptidoglycan (PG) by autolysins. The biogenesis mechanism of BEVs in Gram-positive bacteria remains unclear. OM: Outer membrane. Reproduced from Liang et al. [76], licensed under CC BY 4.0. (**b**) Delivery and associated diseases in human body. BEVs (primarily OMVs), particularly those from periodontal pathogens, can translocate via digestive tract or hematogenous routes (mimicked by oral gavage or injection in models) to distant organs. They are implicated in pulmonary inflammation, atherosclerosis, cancer metastasis, blood–brain barrier (BBB) leakage contributing to Alzheimer’s disease and gastrointestinal inflammation, highlighting the systemic impact of oral–gut crosstalk mediated by BEVs. Reproduced from Chen et al. [77], licensed under CC BY 4.0. (**c**) Host cell entry and pathogenic mechanisms. BEVs enter non-phagocytic host cells via various ways (e.g., lipid raft-associated uptake, clathrin- or caveolin-mediated endocytosis). Inside host cells, they deliver bacterial effectors and immunomodulatory sRNA, or induce mitochondrial damage, and can be detected by host innate immune receptors, triggering pro-inflammatory cytokine production. Finally, internalized BEVs are cleared via host autophagy. Created in BioRender. https://BioRender.com/v6muza7 (accessed on 11 December 2025).

Among these mediating factors, BEVs have emerged as key players in facilitating oral–gut communication. BEVs play roles in both direct and indirect pathways. The direct pathway refers to the disruption of barriers to facilitate microbial migration and colonization, while the indirect pathway involves modulation of the immune system and tumor environments. BEVs are bacteria-derived nanosized, spherical membrane vesicles ranging from 20 to 400 nm in diameter, composed of lipids, proteins, nucleic acids, and other cargos [72]. According to their origins and structures, BEVs are sorted into different types. Gram-negative bacteria produce BEVs in two ways: blebbing and explosive cell lysis. The cell envelope of Gram-negative bacteria consists of the thin cell wall, the outer membrane, the periplasmic space in between, and the inner membrane or cytoplasmic membrane. The most extensively studied BEVs are derived from their outer membrane, referred to as “outer membrane vesicles (OMVs)”, which are rich in outer membrane components. Other detailed classifications regarding BEVs are elaborately introduced in this article [73]. The structural diversity in the cell envelopes results in the differences in biogenesis mechanisms, and cargo composition of their BEVs [74]. These vesicles affect diverse biological processes, including virulence, horizontal gene transfer, export of cellular metabolites, phage infection and cell-to-cell communication, respectively [72]. They interact with host cells by binding to receptors on the cell surface, being internalized through several pathways such as endocytosis and membrane fusion, and releasing their contents into the cytosol [75]. Subsequently, BEVs are cleared by the host autophagy-mediated degradation pathway. The lifecycle of BEVs is illustrated in Figure 3.

Building on this framework, the following section describes the intricate interactions among microbial colonization, host immune responses, neuroimmune signaling cascades, and BEVs, which collectively play a crucial role in modulating the oral–gut crosstalk [17,78].

### 3.1. Direct Mechanisms

#### 3.1.1. Microbial Migration and Colonization

The intra-intestinal pathway of the oral–gut crosstalk primarily refers to the process by which oral microbiota migrates through the digestive tract and colonizes the intestine. Oral bacteria enter the gastrointestinal tract via physiological processes such as saliva swallowing and gastroesophageal reflux. In healthy individuals, barriers such as gastric acid and bile play significant inhibitory roles in preventing oral microbial colonization. Hydrochloric acid (H^+^) secreted by the stomach, along with activation of pepsinogen, effectively kills most oral bacteria swallowed into the stomach, thereby preventing their intestinal colonization [79,80]. Studies showed that reducing gastric acidity through the use of proton pump inhibitors significantly increased the abundance of oral bacteria in the gut, indicating that oral microbiota is more likely to colonize the intestine via the intra-intestinal route when the gastric barrier is weakened [81]. Furthermore, the multilayered defense system of the gut—including the mucus layer, tight junction proteins (e.g., occludin, ZO-1), and intestinal immunity—also plays a critical role in preventing colonization by exogenous bacteria. The specific adaptive traits of microbial strains themselves are decisive for successful colonization of the intestine via the intra-intestinal pathway. Specifically, acid resistance is a prerequisite for microbial survival and intestinal entry. For instance, acid-resistant oral bacteria such as mutans streptococci and *Pg* can be found along the gastrointestinal tract, particularly in the stomach.

Beyond overcoming the aforementioned chemical barriers, oral bacteria must evade biological barriers to colonize the gut, which are imposed by the competitive effects of the resident gut microbiota. Li et al. demonstrated that *Pg* played a pivotal role in competitive interactions within the small intestine against dominant native taxa. In healthy murine small intestine, *Turicibacter* represented the predominant genus (mean relative abundance, 40.40%). Following oral microbiota invasion, the abundance of *Pg* increased while *Turicibacter* decreased sharply to an average of 8.79%. Additionally, *Pg* showed positive correlations with several other dominant oral genera, such as *Streptococcus*, *Enterococcus*, and *Fusobacterium*. Evidence confirms that a healthy gut microbiota constitutes a much more robust barrier than other defense mechanisms [82]. Under disease conditions, the ectopic colonization of oral microorganisms in the gut is markedly increased. Further data indicated that individuals with gastric hypochlorhydria or functional impairments such as IBD exhibit significantly higher rates of oral microbial colonization in the gut. In periodontitis patients, acid-tolerant pathogens like *Pg* are enriched in the oral cavity, enabling them to bypass the gastric barrier, disrupt intestinal homeostasis, and increase gut colonization. Certain bacteria expressing specific adhesins, such as the glucan-binding protein B (GbpB) produced by pathogenic *Streptococcus mutans*, can facilitate enhanced bacterial adhesion to intestinal epithelial cells by disrupting the mucosal barrier and tight junction proteins, thereby exacerbating intestinal inflammation [83]. Oral bacterial components—including endotoxins, peptidoglycan, and DNA—can traverse the intestinal barrier, interacting with TLRs on epithelial cell surfaces and intracellular NOD-like receptors (NLRs), which then induce the production of pro-inflammatory cytokines such as IL-1, IL-6, and IL-8, aggravating intestinal inflammation [84,85]. During periods of inflammation or immunodeficiency, reduced secretory IgA secretion impairs local mucosal immune defenses, increasing the likelihood of microbial translocation. Thus, under conditions such as IBD or periodontitis, even oral bacteria with relatively low abundance can translocate into the gut and actively participate in the inflammatory process via oral–gut immune crosstalk [14].

#### 3.1.2. BEVs-Mediated Pathways

Although bacterial ectopic colonization along the oral–gut crosstalk is well-established, bacterial transit and activity are often impeded by innate physiological barriers, such as gastric acid or the Blood–Brain Barrier [86]. Consequently, successful bacterial colonization and subsequent induction of local dysbiosis usually require a pre-existing compromise in barrier function [87]. Paradoxically, these protective barriers that defend against pathogens also limit the efficacy of probiotic therapies. Therefore, BEVs attract growing attention because they overcome limitations faced by live bacteria, such as enhanced mucus-penetrating ability, modulation of microbiota without colonization, and biosafety properties, exerting essential roles in oral–gut crosstalk [88,89]. Many articles have already summarized that BEVs can migrate to distant organs within the body to exert their effects. Catalan et al. summarized the impact of BEVs produced by periodontitis-associated pathogens (*Pg* and *Fn*) on the gut [90]. Meanwhile, Zhang et al. comprehensively reviewed the role of BEVs derived from both oral and intestinal sources in systemic diseases, such as Alzheimer’s disease and osteoporosis, highlighting the effects of these vesicles on distant organs [91]. BEVs’ association with systemic diseases is shown in Figure 3.

BEVs from oral pathogens can translocate to the gut via the bloodstream or digestive fluids, carrying virulence factors that impair intestinal barrier function, and enhance the bacterial adherence and aggregation on host intestinal cells [92,93,94]. Both in vivo and in vitro experiments have proved that BEVs can significantly reduce the levels of tight junction proteins ZO-1, claudin-1, and occludin, dysregulating the intestinal epithelial barrier integrity, thereby aggravating IBD [95,96,97]. These vesicles typically contain LPS and bacterial proteins derived from the cytoplasm, periplasm, and inner and outer membranes [98,99]. Proteomic investigations have elucidated that *Pg*-derived BEVs harbor various virulence factors, comprising proteolytic enzymes such as gingipains, *Pg* peptidyl arginine deiminase (PPAD), hemagglutinin (HagA), heme-binding protein (HBP35), and fimbrial adhesins (FimA, Mfa1) [100,101]. The protein composition of BEVs differs from that of their parent bacteria [102]. The gingipains are mainly selectively packaged within the BEVs, whose levels on BEVs are three to five times higher than that in their parent bacteria [101,103]. Experiments showed that *Pg*-derived BEVs with gingipains can disrupt the intestinal barrier by damaging Caco-2 cells [95]. As for the BEVs from *Fn*, the virulence factors that function along the oral–gut crosstallk are mainly fusobacterial adhesin (FadA), FomA (an *Fn* outer membrane protein) and fibroblast activation protein 2 (Fap2) [98,104]. Some noncoding RNAs are also enclosed in BEVs, functioning as a virulence factor or its modulator [105,106]. MicroRNAs (miRNAs) in *Fn* BEVs have been shown to contribute to epithelial barrier dysfunction and the aggravation of colitis through the activation of autophagy. In addition, BEVs from *Fn* further accelerate intestinal epithelial cell necroptosis by activating the FADD-RIPK1-caspase 3 signaling pathway, according to Liu et al. [97].

### 3.2. Indirect Mechanisms

#### 3.2.1. Hematogenous Immune Route

It has been elucidated that oral pathogens can colonize the gut and disrupt the intestinal barrier. The colonized bacteria are recognized by colonic immune cells, such as phagocytes, which activate inflammasomes and induce the production of pro-inflammatory cytokines including interleukin-1β (IL-1β), IL-17, and interferon-γ (IFN-γ). Oral pathogens and their products (e.g., LPS), as well as inflammatory mediators, can enter the bloodstream and disseminate systemically, resulting in bacteremia or endotoxemia. Damage to the intestinal barrier facilitates translocation of pathogens and enhances direct interactions with gut immune cells, such as antigen-presenting cells and Th17 cells, further stimulating pro-inflammatory cytokine secretion and exacerbating local inflammation.

In patients with periodontitis, peripheral blood showed significantly increased counts of CD4^+^, CD4^+^CD45RO^+^, IFN-γ^+^ CD4^+^ T and CD8^+^ T cells, CD19^+^CD27^+^ and CD5^+^ B cells, CD14^+^CD16^+^ monocytes, and CD16^+^ neutrophils, with elevated levels of reactive oxygen species (ROS) in neutrophils [107]. Intestinal inflammation is characterized by unconstrained activation of IFNγ-producing CD4^+^ T cells and depletion of regulatory T cells mediated by peripheral Fcγ receptor signaling [108]. Homing of immune cells to the gut promotes monocyte differentiation into intestinal M1 macrophages, which locally stimulate IL-23 expression and promote Th17 cell expansion, thereby aggravating intestinal inflammatory responses. Th17 cells play a critical role in adaptive immunity; during periodontitis, oral pathogen-reactive Th17 cells can migrate to the gut and, upon activation by ectopically colonized oral bacteria, directly induce colitis [17,59]. Additionally, the gut microenvironment itself facilitates enhanced Th17 differentiation. Evidence shows that *P*g aggravates colitis via the gut microbiota-linoleic acid (LA) metabolism-Th17/Treg cell balance axis; *Pg* inhibits the LA pathway in gut microbiota, which normally suppresses Th17 differentiation and promotes Treg cell development, thereby disrupting Th17/Treg equilibrium [109].

Pathological communication between the oral cavity and the gut can activate a multi-organ inflammatory network, with the liver as a typical example. Recently, the oral–gut-liver axis has emerged as a crucial crosstalk pathway in chronic inflammatory diseases. The liver is anatomically adjacent to the gut and engaged in extensive metabolic exchanges and bacterial transport, making it vulnerable to both oral and gut dysbiosis. Under pathological conditions, translocation of oral bacteria into the gut and subsequent intestinal barrier impairment enables LPS entry into the liver via the portal vein, triggering hepatitis. Periodontitis patients display “pro-inflammatory phenotypes” in circulating immune cells (neutrophils and monocytes), releasing excessive ROS and pro-inflammatory mediators, thereby exacerbating hepatic oxidative stress and fibrosis [6,110]. Notably, IL-17 is a well-established pro-fibrotic cytokine involved in the pathophysiology of liver cirrhosis. Moreover, natural killer cell dysfunction in peripheral blood of periodontitis patients may facilitate the persistence of hepatitis B virus and hepatitis C virus infection.

In addition to the direct, forward interactions between oral, gut, and systemic inflammation, the gut can also exert a reverse immunological influence on the oral cavity. Current research has mainly emphasized how intestinal inflammation exacerbrates systemic inflammation and triggers various extraintestinal manifestations, including periodontitis [111,112]. It is speculated that pathogenic T cells activated during intestinal inflammation may migrate to the oral cavity; CCR6^+^ Th17 cells originating from the gut can home to periodontal tissues via the CCL20-CCR6 axis, secrete IL-17 to stimulate osteoclast activation, and drive alveolar bone destruction. In addition, evidence indicates that T cells reactive to Sjögren’s Syndrome Antigen A can be activated by peptides derived from both oral and gut microbiota [113]. This suggests that cross-reactivity between oral and gut microbial peptides and self-antigens may perpetuate autoimmune responses and impair host immune tolerance.

#### 3.2.2. Neuroimmune Route

Accumulating evidence supports bidirectional communication between oral and intestinal tissues via neuroimmune signaling along the oral–gut–brain axis. Disturbances in the oral microbiota can modulate gut microbial composition and immune responses, subsequently affecting central nervous system (CNS) health. Conversely, the brain can regulate gut and oral microenvironments through neuroendocrine and immune pathways. Oral microbiota dysbiosis leads to the translocation of pathogens (e.g., *Pg*) and their metabolites into the gut Via swallowing, disrupting gut microbial homeostasis and inducing intestinal barrier impairment, which manifests as downregulation of tight junction protein expression, mucus layer degradation, and increased intestinal epithelial permeability. The compromised intestinal epithelium releases pro-inflammatory mediators (e.g., TNF-α, IL-1β) and microbiota-derived metabolites (e.g., LPS and SCFAs) that disseminate through the systemic circulation, eliciting systemic inflammatory responses. These inflammatory factors and microbial metabolites can transmit signals via the bloodstream or vagus nerve, cross the blood–brain barrier, and activate microglia and astrocytes, promoting neuroinflammation and β-amyloid (Aβ) deposition, thereby accelerating the progression of neurodegenerative diseases such as Alzheimer’s disease and Parkinson’s disease. Additionally, oral pathogens and their toxins (e.g., gingipains) may also directly affect the brain Via pathways such as the trigeminal nerve [114]. Animal studies demonstrated that transplantation of periodontal pathogens significantly upregulates colonic inflammatory gene expression, downregulates intestinal barrier protein expression, as well as enhances cortical Aβ deposition and glial cell activation; moreover, specific genera (e.g., *Sutterella* are positively correlated with Aβ deposition, while *Lactobacillus* are negatively correlated) are closely associated with pathological parameters of Alzheimer’s disease [115].

On the other hand, the CNS can feedback regulate the intestinal and oral ecology via endocrine mechanisms—including the release of stress hormones through the hypothalamic–pituitary–adrenal axis and catecholamine secretion via the sympathetic-adrenal medullary system—as well as immunomodulatory mechanisms, thus impacting local microecological balance and inflammatory responses [116]. Although specific regulatory mechanisms remain largely undefined, studies indicate that CNS feedback can further exacerbate mucosal immune dysregulation, forming a vicious cycle [114,117].

#### 3.2.3. BEVs-Mediated Pathways

The main role of oral pathogens such as *Pg* and *Fn* and their BEVs in the oral–gut crosstalk is to initiate and promote inflammation. During the inflammatory process, oral BEVs orchestrate the host immune response, modulate intestinal barrier function and facilitate the dissemination of bacterial components. BEVs are able to reach the intestine after intragastric administration without being degraded, and can exacerbate colitis in colitis mouse models [96]. In addition to disrupting the intestinal epithelial barrier, BEVs also regulate gut epithelial cell innate immunity by activating TLR2, TLR4, NOD1 and NOD2, thereby inducing the activation of NF-κB and MAPK signaling pathways, which drives the production of pro-inflammatory cytokines (IL-8 and TNF) and initiates inflammation [92,118,119]. *Fn*-derived BEVs also significantly promote the differentiation of pro-inflammatory macrophages and cell necroptosis in both the oral and intestinal barriers [97,120]. While BEVs from *Pg* can compromise neutrophil function to evade immune clearance. They selectively coat and activate human neutrophils, inducing degranulation and suppressing neutrophil antimicrobial activity via gingipain-mediated degradation of antibacterial components, especially LL-37 and myeloperoxidase (MPO) [121]. Additionally, they enhance the pro-inflammatory effect of the bacteria by inducing tolerance in monocytes [122].

In the field of oncology, *Fn* has attracted significant attention due to its role in promoting the initiation and progression of oral squamous cell carcinoma and CRC [123,124,125]. The functions of its BEVs have also gradually been revealed. *Fn*-derived BEVs are significantly enriched in both clinical CRC tissues and CRC-bearing mouse models. Oral or intravenous administration of *Fn*-derived BEVs can passively accumulate in tumor tissues through the enhanced permeability and retention effect, followed by their entry into CRC cells through membrane fusion in an acidic microenvironment. The virulence factors through which these BEVs function along the oral–gut crosstalk are mainly FadA, FomA and Fap2 [98,104]. Within the tumor niche, BEVs deliver the adhesin FomA to the surface of neoplastic cells, where it interacts with the bacterial surface protein FN1441 to promote *Fn* auto-aggregation and colonization [126]. In addition, BEV-associated virulence proteins (including homologous autotransporters of Fap2 and MORN2 domain-containing effectors) are upregulated under acidic conditions, which may promote the progression of CRC by enhancing bacterial adhesion, immune evasion, or directly activating host cell signaling pathways [98]. During tumorigenesis, *Fn* enhances oncogenic potential by delivering FadA, which can activate the Wnt/β-catenin signaling pathway, driving oncogene expression and tumor cell proliferation in colorectal tissues [127,128]. Given that BEVs contain a substantial amount of FadA, it is plausible that this is one of the mechanisms underlying the function of BEVs in CRC. However, there is currently no direct experimental evidence to clearly confirm this. BEVs carrying FomA and FadA may also serve as novel targets for therapeutic intervention in oral and colorectal cancer.

In conclusion, BEVs of oral pathogens disrupt the intestinal barrier, induce immune responses and promote the development of inflammation and tumors. Identification of these BEVs in the oral cavity serves as evidence of periodontitis [129]; meanwhile, their presence in the gut can potentially be linked to the diagnosis of IBD and colorectal cancer, although their diagnostic sensitivity and specificity require further validation. Given their central role, these BEVs are anticipated to become promising targets for managing gut-related inflammatory diseases and cancers.

Oral–gut microbial interaction mechanisms and related diseases described above are summarized in Table 2.

**Table 2:** Summarization of oral–gut microbial crosstalk mechanisms.

Mechanism Category	Mediating Route	Key Mediators/Factors	Effects	Associated Diseases	References
Direct mechanisms	Microbial Migration and Colonization	Oral bacteria	Disrupt microbial homeostasis	IBD, periodontitis	Lourenço, T.G.B. et al. [42]
	BEVs- Mediated Pathways	BEVs from oral pathogens (*Pg*, *Fn*), containing virulence factors	Increase epithelial barrier permeability by degradation of the tight junction protein.	IBD	Nonaka, S. et al. [95]
			Modulate autophagy via the miR-574-5p/CARD3 axis.	IBD	Wei, S. et al. [96]
			Accelerate intestinal epithelial cells necroptosis by activating FADD-RIPK1-caspase 3 signaling.	IBD	Liu, L. et al. [97]
Indirect mechanisms	Hematogenous immune route	Immune cells (Th17, Treg, monocytes/macrophages), cytokines (IL-1β, IL-17, IFN-γ), microbial metabolites (linoleic acid)	Trigger local inflammatory response	Liver cirrhosis, Hepatitis	Mester, A. et al. [110]
	Neuroimmune route	Inflammatory mediators (TNF-α, IL-1β), microbial products (LPS, SCFAs), Vagus nerve	Promote neuroinflammation via circulation/neuronal pathways	Alzheimer’s disease, Parkinson’s disease.	Sansores-España, L.D. et al. [114]
	BEVs- Mediated Pathways	BEVs from oral pathogens (*Pg*, *Fn*), containing virulence factors	Promote pro-inflammatory response by binding TLR2 and inducing the NF-κB signaling pathway.	IBD	Martin-Gallausiaux, C. et al. [92], Wei, S. et al. [96], Liu, L. et al. [97]
		BEVs from oral pathogens (*Fn*), containing virulence factors	Present a target for bacterial adhesion by transferring to CRC cell surfaces.	CRC	Zheng, X. et al. [126]

IBD: Inflammatory bowel disease; CRC: Colorectal cancer; *Pg*: *Porphyromonas gingivalis*; *Fn*: *Fusobacterium nucleatum*; BEVs: Bacterial extracellular vesicles; LPS: Lipopolysaccharides; SCFAs: Short-chain fatty acids.

## 4. Therapeutic Application of Bacteria and Their BEVs Based on the Oral–Gut Microbial Crosstalk

Owing to BEVs’ nanoscale size and the biological molecules such as key proteins and RNAs they carry, BEVs possess inherent advantages in penetrating physiological barriers and exerting effects similar to those of their parent bacteria. For BEVs derived from pathogenic bacteria, this characteristic can exacerbate microenvironmental disorders and accelerate disease progression. In contrast, for BEVs derived from probiotics, this ability can be developed into a novel therapeutic strategy. The core advantage of using BEVs as a therapeutic approach lies in their ability to precisely deliver functional molecules without biosafety risks posed by live bacterial infection and uncontrolled proliferation. This section will review the applications of probiotics in treating diseases along the oral–gut crosstalk and summarize the emerging therapeutic applications of BEVs, discussing the prospects for their future development in oral–gut crosstalk.

Gut probiotics and their BEVs have been widely studied for their roles in regulating intestinal microbial homeostasis and alleviating inflammation [22,130]. However, their application in oral treatment has not yet been fully discussed. *Akk* is a representative gut probiotic, and its anti-inflammatory properties along the oral–gut crosstalk have recently been demonstrated. As a Gram-negative anaerobic bacterium that colonizes the intestinal mucus layer, *Akk* was first isolated from the feces of healthy adults in 2004. *Akk* accounts for up to 4% of the gut microbiota and is regarded as the “next-generation probiotic” because it can maintain intestinal barrier integrity and immune homeostasis, as well as improve metabolism [131,132]. The roles of *Akk* in the gut–brain axis, the oral–gut crosstalk, and the treatment of various infectious diseases have also gradually gained attention [133]. Clinical studies have shown that patients with a decline in *Akk* correlate with periodontitis and IBD, suggesting the potential therapeutic value of *Akk* in these diseases [134,135]. In vivo studies have shown that *Akk* not only mitigates intestinal inflammation but also reduces tissue damage and bone loss in periodontitis, proving that *Akk* is effective in treating periodontitis caused by *Pg* and *Fn*. In a *Pg*-induced experimental periodontitis model conducted by Huck et al., a significant reduction in alveolar bone loss was measured after therapeutic administration of *Akk* [136]. Another in vivo experiment by Song et al. found that compared with the group inoculated with *Fn*, the group that inoculated with *Akk* and *Fn* had a higher species richness of the bacterial community, as well as reduced alveolar bone loss and tissue inflammation. *Akk* exerts such an effect mainly through several key mechanisms. It suppresses the growth and virulence factor expression (e.g., FadA, Fap2, Aid1, FomA, and CmpA) of *Fn* in co-culture, thereby reducing its adherence to and invasion of gingival epithelial cells. The oral community structure modulated by *Akk* has a higher species richness, higher abundance of beneficial bacteria, and reduced colonization of pathogens including *Fn*, *Prevotella*, *Treponema*, and *Campylobacter* [137]. Additionally, *Akk* administration promotes the expression of intercellular adhesion molecule-1 (ICAM-1) and ZO-1 in gingival epithelial cells to enhance the barrier function [138]. *Akk* modulates the immune response by promoting the secretion of IL-10 while downregulating pro-inflammatory cytokines (e.g., TNF-α, IL-1β, IL-6, and IL-8) through the TLR/MyD88/NF-κB signaling pathway [137]. This immune regulation is further enhanced by polarizing macrophages towards an anti-inflammatory phenotype. It is worth mentioning that a pili-like protein of *Akk*, Amuc_1100, has also shown its effecacy in relieving periodontal inflammation [139]. Moreover, *Akk* inhibits *Pg*-induced osteoclast activation and inflammation, highlighting their potential to regulate bone metabolism via the oral–gut crosstalk [136].

Meanwhile, BEVs derived from *Akk*, which carry a complex proteome enriched in membrane and cytoplasmic proteins involved in metabolic and anti-inflammatory pathways, show potential for therapeutic applications in inflammatory diseases [140]. Studies confirm that BEVs derived from *Akk* can blunt dextran sodium sulfate (DSS)-induced acute colitis, with effects similar to giving *Akk* through oral administration alone in many aspects, including weight regain, colon length and inflammatory cell infiltration of colon wall [141]. Wang et al. proposed that BEVs from *Akk* alleviate IBD through three interconnected mechanisms: restoring gut microbiota homeostasis by promoting beneficial bacteria proliferation via membrane fusion; inducing IgA responses through translocation to Peyer’s patches and immune cell activation; and enhancing intestinal barrier integrity through the stimulation of tight junction protein expression and mucus production [22].

**Figure 4:**
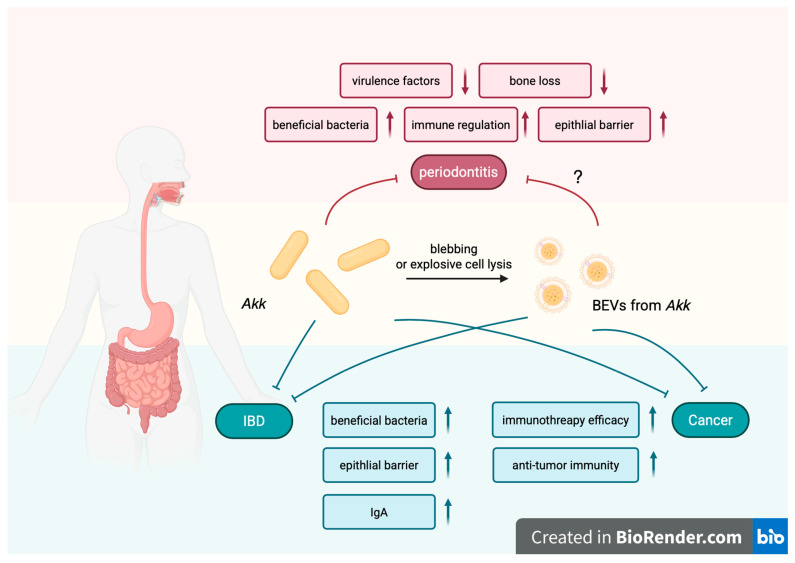
Schematic overview of the regulatory roles of *Akkermansia muciniphila* (*Akk*) and its bacterial extracellular vesicles (BEVs) in oral–gut crosstalk-related diseases. *Akk* and its BEVs promote the growth of beneficial microbial species while reducing the expression of virulence factors from oral pathogens. They enhance epithelial barrier integrity and contribute to immune homeostasis under inflammatory conditions. In cancer, they improve the efficacy of immunotherapy and stimulate anti-tumor immunity. Areas requiring further investigation are indicated with question marks (“?”). Created in BioRender. https://BioRender.com/gxdsdcw (accessed on 19 October 2025).

In addition, BEVs from *Akk* exhibit significant potential in the treatment of oral pathogen *Fn*-associated tumors and cancers. In a murine model of CRC, they improved the efficacy of immunotherapy targeting PD-1 [22]. This immunomodulatory effect involved the activation of CD8^+^ T cells and an increase in M1-like macrophages [142]. Furthermore, engineered *Akk*-derived BEVs were developed as nanocarriers of *Atox1* siRNA and elesclomol, which enhanced copper-induced cell death and activated anti-tumor immunity. They showed excellent efficacy in models of subcutaneous breast cancer and orthotopic rectal cancer [143]. Collectively, these findings highlight the multifaceted therapeutic potential of BEVs from *Akk* in *Fn*-associated cancers, positioning them as a promising adjuvant for enhancing existing cancer treatments. Beyond oncology, *Akk*-derived BEVs show promise in bone regeneration. They exhibit the capacity to accumulate within bone tissue, where they effectively attenuate ovariectomy-induced osteoporosis. This protective effect is mediated through the enhancement of osteogenic activity and the suppression of osteoclast formation [144]. Although there is no direct evidence of *Akk*-derived BEVs functioning in the oral cavity, the osteogenic and anti-osteoclastogenic properties suggest significant potential for their direct application, or use as a targeted delivery vehicle, in the treatment of periodontal disease. The functions of *Akk* and its BEVs in regulating oral–gut crosstalk-related diseases are illustrated in Figure 4.

Several other gut bacterial species have demonstrated therapeutic potential against oral inflammation as well. In the context of periodontitis, they maintain periodontal health by inhibiting the growth and virulence of pathogenic species in the oral microbiota, modulating the immune response, and promoting tissue healing processes [18]. Extensive reviews have summarized on this point. Certain gut or oral–gut-shared probiotics, such as members of the *Lactobacillus*, *Streptococcus*, and *Bifidobacterium* genera are frequently studied as adjunctive therapies in periodontitis. Their efficacy on clinical parameters is comprehensively summarized in the review by Baddouri et al. [145]. However, it is also mentioned that while probiotic therapy may demonstrate initial and short-term benefits for a few months, its long-term efficacy remains questionable due to subsequent regression and the lack of sustained advantage over control groups [18,146,147]. Moreover, some results reported from clinical studies on the efficacy of *Bifidobacterium* in preventing and treating oral microbial diseases are conflicting and controversial. This is mainly reflected in changes in *Streptococcus mutans*, with some studies reporting reductions and others showing no significant effect [146]. *Streptococcus mutans* is one of the crucial bacteria causing dental caries and can also affect periodontal health indirectly [148]. The possible risks that the consumption of probiotics can cause in individuals with compromised immune systems should also be considered.

In recent years, BEVs derived from these probiotics (mainly members of the *Lactobacillus* and *Bifidobacterium*) have also demonstrated significant therapeutic potential. Although most current research focuses on intestinal and systemic diseases, BEVs present a potential solution to certain limitations of probiotic therapies owing to their superior stability. In terms of intestinal inflammation, many animal experiments have shown that BEVs of the *Bacteroides* and *Bifidobacterium* genera can alleviate DSS-induced colitis. It is noteworthy that the BEVs of *Bacteroides acidifaciens* exerted a more pronounced alleviating effect on colitis in a mouse model than their parent bacteria, suggesting that BEVs may have higher targeting and delivery efficiency. These BEVs mainly reduce intestinal inflammation by regulating intestinal immunity; for example, *Bacteroides fragilis* BEVs interact between polysaccharide A on the vesicle surface and TLR2 on dendritic cells (DCs), activating intracellular signaling to promote an anti-inflammatory response, which is implicated in the prevention of colitis [149]; *Bacteroides thetaiotaomicron* BEVs are able to induce IL-10 expression in colonic DCs and IL-6 and CD80 expression in blood-derived DCs [150]. In addition, BEVs can specifically regulate immune cell populations in the gut, affecting the Th17/Treg balance. A strain of *Bifidobacterium bifidum* was found to produce BEVs capable of promoting the differentiation of functional Treg and the release of the anti-inflammatory cytokine IL-10 [151]. In vitro and in vivo study using BEVs from *Lactobacillus paracasei* found that they were able to augment the endoplasmic reticulum stress pathway, increasing anti-inflammatory factors and decreasing pro-inflammatory factors to alleviate colitis [152]. As proposed in a review by Melo-Marques et al., another possible mechanism is that BEVs carry glycosidases and proteases, which can degrade polysaccharides and proteins and provide nutrients for themselves and other commensals. This “public goods” supply mechanism helps maintain the balance of the microbial community [89]. In terms of bone metabolism, in addition to *Akk* BEVs that can help protect against osteoporosis, Wang et al. found that *Proteus mirabilis* BEVs induce mitochondrial apoptosis to inhibit the differentiation and function of osteoclasts, significantly ameliorating bone loss in osteoporosis and rheumatoid arthritis animal models [153]. Similarly, *Lactobacillus salivarius* helps mitigate inflammation-mediated bone loss by repairing the intestinal barrier. It can also promote osteogenesis and inhibit osteoclast activity by secreting BEVs, which can be transported to bone tissue [154]. Other mechanisms by which oral and gut BEVs regulate bone metabolism include regulating immune balance and mediating the production of metabolites such as SCFAs, as discussed in detail in a review by Liang et al. [76]. Crucially, these systemic signals ultimately act on osteoblasts, where they activate the core mechanisms responsible for osteogenic differentiation. This process is intrinsically dependent on extensive metabolic reprogramming—particularly in glucose metabolism and mitochondrial function—to meet the biosynthetic and energy demands of bone matrix synthesis [155]. Accordingly, further investigation into how BEVs systemically modulate such local cellular metabolic programs is necessary.

While BEVs exert anti-inflammatory and tissue repair effects via their intrinsic components, they can also be engineered to serve as drug delivery vehicles or to enhance tissue targeting, thereby addressing the functional limitations of natural bacteria or vesicles. One primary strategy is genetic engineering. Through plasmid-based and CRISPR-Cas9-based systems, specific genes can be overexpressed or knocked out to enrich or deplete specific effectors. Carvalho et al. engineered BEVs derived from *Bacteroides thetaiotaomicron* that stably expressed and delivered Keratinocyte growth factor-2 (KGF-2) into the gastrointestinal tract, providing protection against tissue inflammation and injury. In colitis mouse models, these engineered BEVs decreased disease severity and promoted the repair and recovery of the damaged epithelial barriers, while requiring doses one to two orders of magnitude lower than conventional daily injections to achieve a comparable reduction in colonic pathology, demonstrating the superior delivery efficiency of engineered BEVs [156]. In the context of osteogenesis, bone morphogenetic protein-2 (BMP-2) and vascular endothelial growth factor (VEGF), both known to promote the osteoblast differentiation from bone marrow mesenchymal stem cells, have been successfully overexpressed in bacteria and represent promising candidates for the development of therapeutic BEVs aimed at bone regeneration [157,158]. Liu et al. constructed a recombinant probiotic, *Escherichia coli* Nissle 1917-pET28a-ClyA-BMP-2-CXCR4, in which BMP-2 and CXCR4 were overexpressed in fusion with BEVs surface protein ClyA. BEVs isolated from this strain exhibited great bone targeting ability in vivo [159]. The encapsulation of specific substances into BEVs can also be achieved via techniques such as electroporation, sonication, and extrusion [160]. Beyond cargo loading, through surface modification, BEVs can be engineered to display specific targeting molecules (e.g., ligands or antibodies), which enables precise cell recognition and binding to enhance treatment efficacy while minimizing side effects [161,162]. Liu et al. developed engineered BEVs from *Lactobacillus rhamnosus* by anchoring bone-targeting peptides on the membranes, which endowed them with the ability to deliver intrinsic miRNA to the bone microenvironment. The engineered BEVs exhibited robust bone-targeting capabilities, promoted osteogenic differentiation and mineralization, inhibited the formation of osteoclasts, and effectively ameliorated osteoporosis [163]. The precise targeting, immunomodulatory and tissue repair capabilities demonstrated by engineered BEVs in bone metabolism have shown promising results, providing a mechanistic reference for their application in oral–gut crosstalk. Given the shared mechanisms such as inflammation regulation and microbial-host interactions, the delivery strategies based on BEVs are expected to be transferred to the intervention of oral–gut-related diseases. This could involve targeting the local immune microenvironment in the oral cavity or intestine, delivering immunomodulators or drugs, thereby enabling coordinated therapy for oral–gut disorders.

## 5. Current Challenges and Controversies in Oral-to-Gut Microbial Translocation and BEV Research

Although the research and application prospects of oral–gut interactions and related BEVs are broad, their scientific interpretation and clinical translation still face multiple challenges. A major difficulty lies in establishing clear causal relationships. Owing to the complex and bidirectional association between IBD and periodontitis, most current evidence is derived from cross-sectional analyses of patients’ microbial profiles, making it difficult to determine whether oral microbial or BEV translocation to the gut actively contributes to intestinal inflammation or merely reflects compromised barrier function. These uncertainties are further exacerbated by methodological limitations in microbiota research. Commonly used 16S rRNA sequencing primarily reports relative abundance, which may obscure true microbial population changes in the highly inflammatory and fluctuating environment of periodontitis or IBD. Consequently, the magnitude and functional relevance of microbial changes may be misinterpreted. Addressing these limitations will require well-designed longitudinal and cohort studies to clarify the role of oral–gut crosstalk in the coevolution of intestinal and periodontal diseases.

Beyond the traditional focus on bacterial abundance and function, the role of BEVs should also be considered. Given the intricate interactions between oral–gut microbiota and the host immune system, distinguishing the individual effects of BEVs from live bacteria in vivo is highly challenging. Furthermore, as the mechanisms summarized in Section 3 of this review, the oral–gut crosstalk is closely linked with multiple organs and systems throughout the body. Therefore, any therapeutic intervention targeting it using probiotics or their BEVs must take their potential systemic dissemination into consideration. Studies show that BEVs can enter the circulatory system and penetrate various physiological barriers, including the blood–brain barrier and the placental barrier [164,165], which may lead to their accumulation in non-target organs and elicit unforeseen adverse effects. Consequently, a deeper understanding of these complex interaction networks and evaluation of the systemic impacts of interventions represent major challenges for future research on the oral–gut crosstalk.

Apart from the risk of transmission across physiological barriers, there remain several other challenges before BEVs can be translated into clinical practice. First, the intrinsic heterogeneity of BEV cargo, shaped by environmental conditions such as pH, vesicle size and bacterial growth phase, complicates reproducibility. Addressing this requires advanced isolation and sorting technologies with improved yield, purity, and the ability to separate contaminants. Second, the immunogenicity of BEVs must be precisely modulated. Engineering BEVs to enhance safety is a priority, with the particular need to reduce or eliminate immunogenic components such as outer membrane protein A (OmpA) or excessive LPS. Third, the long-term stability of BEVs during storage and in vivo application directly impacts their therapeutic consistency. In particular, differences between the oral and gut luminal pH necessitate reliable and well-validated formulation strategies. Optimal dosing parameters, including administration routes and frequency for specific diseases, remain undefined and must be established through controlled preclinical and clinical studies, with careful consideration of interspecies differences in microbiota composition between humans and commonly used animal models. Finally, considering all of the above, achieving scalable, cost-effective, and standardized industrial production represents a fundamental manufacturing bottleneck that must be solved through innovations in bioprocessing.

## 6. Conclusions and Future Perspectives

The oral–gut microbial crosstalk deepens our understanding of the impact of the oral microenvironment on systemic health and disease, especially with respect to gut health and disorders. This inter-organ connection emphasizes the important role of increased abundance and virulence of oral disease-associated bacteria colonizing the gut in exacerbating IBD, particularly when there is a reduction in intestinal barrier resistance. Conversely, systemic inflammation and immune dysregulation associated with IBD can disrupt the homeostasis of the oral microenvironment, thereby aggravating oral inflammation. Among these interplays, the coordination among microbial communities is essential, as it involves the microbial balance of the oral cavity and gut, the integrity of the intestinal and oral epithelial barriers, and host immune regulation. Therefore, the oral and gut microbiota have become promising targets for the management of systemic diseases. Our review focuses on the oral–gut crosstalk, its underlying mechanisms and derived therapeutic potentials, based on representative oral and gut disorders, namely periodontitis and IBD.

Apart from bacteria themselves, their BEVs are of growing importance in oral–gut interactions. We summarize the pathogenic effects of oral pathogen-derived BEVs, and discuss the potential therapeutic applications of probiotic-derived BEVs. However, compared with the extensive studies examining their effects in treating IBD, most studies investigating BEVs in periodontitis have focused on oral pathogen-derived BEVs, rather than probiotic-derived ones for therapeutic purposes. Compared to probiotics, their BEVs offer distinct advantages in terms of biosafety, as they influence microbial community structure without causing uncontrolled colonization and infection, endowing BEVs with high translational potential. Given that the current prevalence of periodontitis remains high, systemic regulation mediated by BEVs may represent a new treatment option.

Rapidly advancing research on oral–gut crosstalk holds immense promise for elucidating inter-organ interactions in health and disease, and for highlighting the vital role of the oral cavity as a gateway to systemic homeostasis. Our growing understanding of the oral–gut crosstalk will facilitate the development of innovative therapeutic strategies for oral and gut disorders, as well as for systemic diseases.

## Data Availability

No new data were created or analyzed in this study. Data sharing does not apply to this article.
